# Supplementary material for: GLIS1 in Cancer-Associated Fibroblasts Regulates the Migration and Invasion of Ovarian Cancer Cells
Source: Int J Mol Sci. 2022 Feb 17;23(4):2218. doi: 10.3390/ijms23042218 (PMC8874490; doi:10.3390/ijms23042218)
Supplement: Supplementary file 1 [file ijms-23-02218-s001.zip › supplementray_IJMS_R1.pdf]

International Journal of *Molecular Sciences*

Supplementary Materials

GLIS1 in Cancer-Associated Fibroblasts Regulates the Migration and Invasion of Ovarian Cancer Cells

Mi Joung Kim [1,†], Daun Jung [2,†], Joo Youn Park [1], Seung Min Lee [1] and Hee Jung An [2,*]

[1] Institute for Clinical Research, CHA Bundang Medical Center, CHA University, Seongnam 13496, Korea; mjkim7845@naver.com (M.J.K.); jypark@naver.com (J.Y.P.); smin0515@chamc.co.kr (S.M.L.)
[2] Department of Pathology, CHA Bundang Medical Center, CHA University, Seongnam 13496, Korea; jhd2800@hanmail.net
* Correspondence: hjahn@cha.ac.kr; Tel.: +82-31-780-5439
† These authors contributed equally to this work.

Table S1. List of primer sequences used for RT-PCR analysis.

Gene Name		Sequence (5′→34)
FAP	Forward	GTTATTGCCTATTCCTATTATG
	Reverse	GTCCATCATGAAGGGTGGAAA
DCHS1	Forward	CTGAAACACGGTTGGTGCTG
	Reverse	CCCCAGTTGCCACTGATGAT
GLIS1	Forward	CCATTCAGAGACTGGCGTGA
	Reverse	TCTGGTGCACAGTTTGGTGA
Cytokeratin 7	Forward	TGAGATCGACAACATCAAGAAC
	Reverse	CGGATGGAATAAGCCTTCAG
GAPDH	Forward	GGACCTGACCTGCCGTCTAG
	Reverse	GGCCATGTGGGCCATGAGGTC

Table S2. List of antibodies used for Western blot analysis.

Name	Catalog No.	Company
FAP	ab53066	Abcam
DCHS1	ab130092	Abcam
GLIS1	GTX117785	GeneTex
β-actin	sc-47778	Santa Cruz Biotechnology

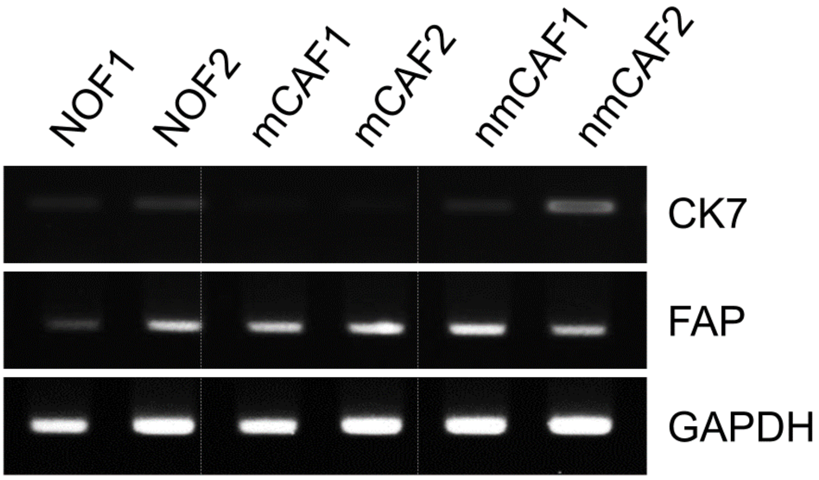

Figure S1. Purity of the isolated CAFs. The mRNA expression of CK7 and FAP in CAFs isolated from human ovarian carcinoma tissues was detected by using RT-PCR. GAPDH was used as a loading control.

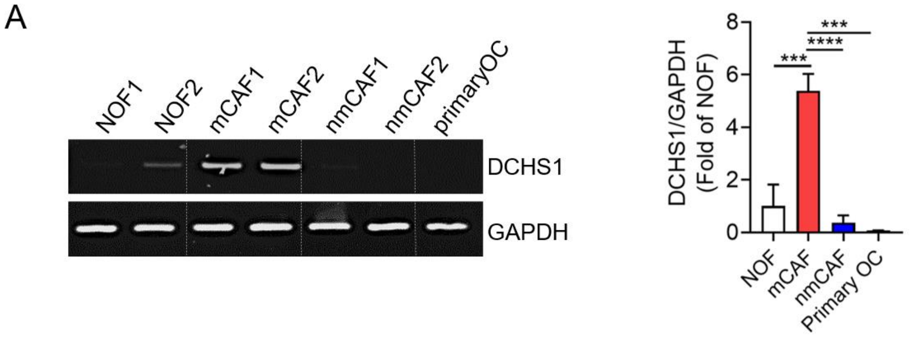

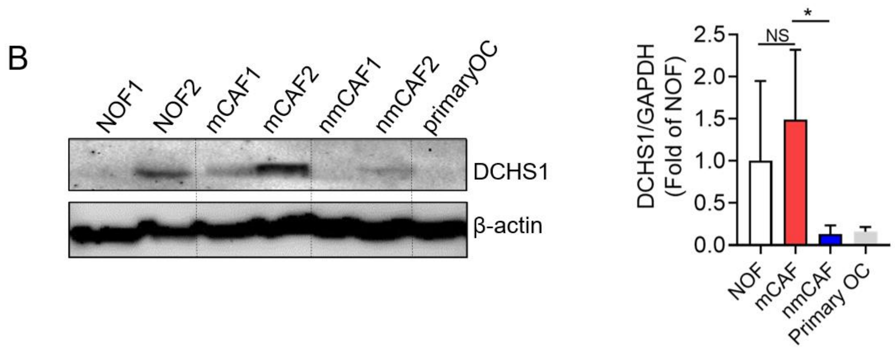

Figure S2. Expression of DCHS1 mRNA and protein in NOF, mCAF, nmCAF and carcinoma cells. (**A**) mRNA expression levels was examined using RT-PCR. The quantification of relative mRNA levels was normalized to GAPDH. (**B**) Immunoblot analysis of DCHS1 in NOF, mCAF, nmCAF and carcinoma cells. The ratio of the intensity of protein bands relative to that of β-actin was calculated. Bar graph represents the relative protein expression of DCHS1. Each experiment was performed in triplicate. Data are represented as mean ± SD. Statistical analysis was performed using an unpaired t-test (*$p < 0.05$, *** $p < 0.001$, **** $p < 0.0001$).

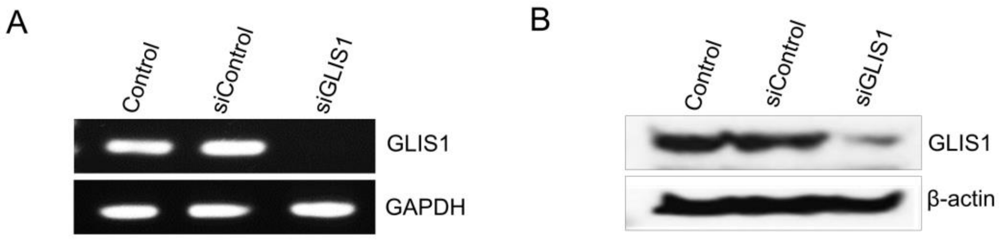

Figure S3. Knockdown GLIS1 in CAFs**.** Efficiency of siRNA treatment of GLIS1 on CAFs. The control sample transfected with neither siGLIS1 nor siRNA control. Gene knockdown efficiencies were obtained using RT-PCR (**A**) and western blot (**B**).
